# Long-term follow up of surgical management of blood blister-like aneurysms at non-branching sites of the internal carotid artery

**DOI:** 10.1097/MD.0000000000033371

**Published:** 2023-03-24

**Authors:** Hyeon-Ju Kim, Jong-Myong Lee

**Affiliations:** a Department of Neurosurgery, Jeonbuk National University Hospital and Medical School, Jeon-Ju, South Korea.

**Keywords:** blood blister like aneurysms, carotid stenosis, subarachnoid hemorrhage

## Abstract

To describe long-term follow-up of 25 patients who presented with subarachnoid hemorrhages due to blood blister-like aneurysms (BBAs) treated with direct clipping or clip reinforcement with or without direct neck repair. Between June 1993 and July 2009, 25 consecutive patients with ruptured BBAs of the supraclinoid internal carotid artery were retrospectively reviewed. The mean age of patients was 39.5 ± 11.3 years. The mean duration of clinical follow-up was 128.9 months (range, 85–196 months). All aneurysms were located in the supraclinoid portion of the internal carotid artery. The mean aneurysm diameter was 4.04 ± 1.3 mm on intra-operative microscopic field. Tearing of the aneurysmal neck during dissection occurred in 8 (32%) patients. Six of 7 patients with neck tearing underwent direct neck repair. Surgeons treated aneurysms via direct clipping with a Bemsheet^®^ in 5 (20%) patients or by clip reinforcement with a silicone sheet in 20 (80%) patients. Clinical outcomes were favorable (modified Rankin Scale [mRS]: 0–2) in 21 (84%) of 25 patients. Four (16%) patients had an unfavorable outcome (mRS: 3–6). The patient with severe disability (mRS: 4) was treated with clip reinforcement and direct neck repair. Mild stenosis, moderate stenosis, and total occlusion of the parent artery were confirmed in 10 (40%) patients, 6 (24%) patients, and 1 (4%) patient, respectively. Although surgical treatment of BBAs was associated with varying degrees of parent vessel patency loss, long-term follow-up results for more than 10 years showed that direct surgical clipping or clip reinforcement with a silicone sheet appeared to be a curative surgery.

## 1. Introduction

Very small aneurysms originating from non-branching sites of the supraclinoid portion of the internal carotid artery (ICA), so called “blood blister-like” aneurysms (BBAs), have thin walls that can easily undergo premature rupture during surgery. They are associated with post-operative rebleeding.^[1–8]^ Many treatment options have been introduced for surgical treatment of BBAs, such as wrapping, clipping, direct neck suture, encircling clip, and ICA trapping with or without bypass surgery.^[1,9–14]^ Recently, endovascular treatments including primary coil embolization, stent-assisted or -only procedures, and endovascular trapping have been reported.^[15–26]^ Endovascular treatment has been proven to be relatively effective in preventing early rebleeding. However, the total obliteration rate of aneurysms is lower than that achieved by direct surgery.^[4,7,8,16–18,22,27]^ Morbidity and mortality rates, aneurysm growth, and rebleeding are very high in all treatment modalities.^[1,2,6,24,28,29]^ Despite developments in the treatment of acute aneurysmal subarachnoid hemorrhage (SAH), the case fatality rate has remained stable. Outcomes of patients with BBAs have been a technical challenge with poorer clinical outcomes than those of patients with saccular aneurysms regardless of treatment modality.^[1,5,6,11,30]^

The management of ruptured BBAs remains controversial. Detailed long-term outcome data are still lacking. The objective of the present retrospective study was to determine clinical outcomes of a series of 25 patients presented with SAHs due to BBAs who were treated with direct clipping or clip reinforcement with or without direct neck repair after a long-term follow-up.

## 2. Clinical materials and methods

### 2.1. Patient population

Between June 1993 and July 2009, 25 patients with ruptured BBAs of the supraclinoid ICA were consecutively treated at our institution. The study protocol was approved by our Institutional Review Board. Informed consent was waived for relatives of the deceased. Patients with unruptured or incidental aneurysms were excluded from this study. Hospital records and cerebral angiography (CAG) findings were retrospectively reviewed.

### 2.2. Clinical evaluation

All patients underwent a detailed analysis consisting of clinical status (Hunt and Hess scale), radiologic findings (Fisher scale), angiographic findings, and surgical treatment methods. The outcome measure was the modified Rankin Scale (mRS) at discharge, at 6 to 12 months after SAH, and during subsequent hospitalizations or consultations in the outpatient clinic. Furthermore, the outcome was dichotomized as favorable (mRS score of 0–2) or unfavorable (mRS score of 3–6).^[31]^ Procedure-related morbidity was defined as any permanent deficit related to treatment. At the final follow-up, overall morbidity/mortality, mRS score, and neurologic deficits were evaluated.

### 2.3. Radiologic study

**Figure 1.**
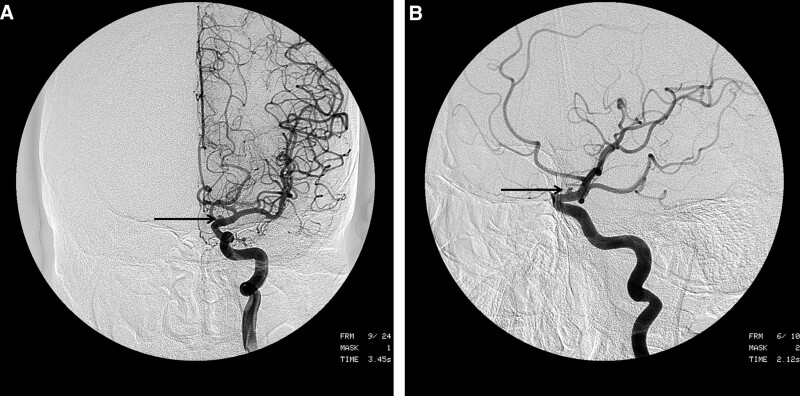
(A and B) Cerebral angiographic studies demonstrating a broad-based faint bulge on the dorsal wall of the left ICA. ICA = internal carotid artery.

Patients underwent radiologic evaluation at our hospital by means of brain computed tomography (CT) and brain CT angiography shortly after arrival. All patients underwent 4-vessel angiography. Before 2001, conventional CAG was the diagnostic tool. After 2001, the diagnostic tool changed to digital subtraction angiography (DSA) (Fig. 1A and B). Based on preoperative CT findings, patient’s condition was scored according to the Fisher scale. All patients underwent postoperative DSA. Postoperative supraclinoid stenosis of the ICA was categorized as follows: normal, mild stenosis (<29%), moderate stenosis (30%–69%), severe stenosis (70%–99%), or total occlusion (99%–100%) as detailed in the North American Symptomatic Carotid Endarterectomy Trial criteria.^[32]^

### 2.4. Surgical technique

**Figure 2.**
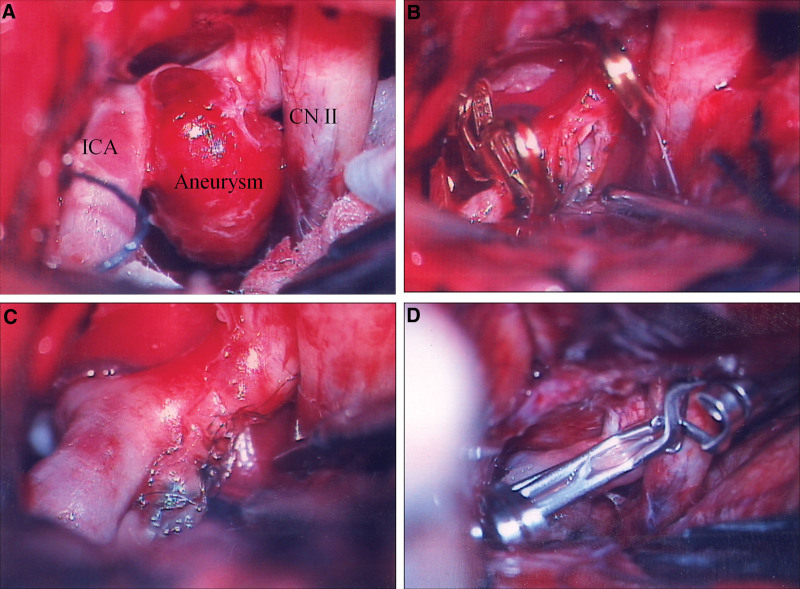
(A) Intraoperative photograph showing an aneurysm covered with a blood clot. (B) Intraoperative photograph demonstrating direct repair of the arterial wall with 8-0 nylon after temporary trapping. (C) Successful arterial suturing of the torn neck. (D) Intraoperative photograph showing reinforcement of the sutured vessel wall with application of combined wrapping and clipping.

The operation was performed by an expert single neurovascular surgeon, who performed an average of 70 cases aneurysm clipping per year for 16 years. All patients underwent standard pterional or orbitopterional craniotomy (Fig. 2A). The Sylvian fissure was widely opened. The ICA, anterior cerebral artery, and middle cerebral artery were exposed. Intra-dural drilling of the anterior clinoid process was performed if proximal control of the ICA was not achieved in the operative field. The clot that covered the aneurysm was removed carefully. Proximal and distal portions of the aneurysm, posterior communicating artery, and anterior choroidal artery were completely dissected. Subpial dissection of frontal or temporal lobes was performed at the final stage immediately before applying an aneurysm clip.

In the case of direct neck clipping, the thin wall around the aneurysm and the aneurysm itself were covered with a cotton pad (Bemsheet^®^; Kawamoto, Osaka, Japan) attached by fibrin glue. Clipping was then performed. Blood velocity was checked using an intraoperative microvascular Doppler. When premature rupture due to tearing of the neck during dissection occurred before clipping the aneurysm, the aneurysm or ICA was trapped with temporary clips. Direct suture of the wall was then performed with 8-0 nylon.

First, the ICA and aneurysm were circumferentially wrapped using a silicone sheet (medical grade silicone sheet, size#20-05; Bioplexus Corporation, Kingman, AZ). Two leaves of the sheet were held with a long micropipette and a 90-degree clip was applied on the sheet parallel to the ICA or the arterial wall beyond the aneurysm. When branches or perforator from the ICA and the ICA appeared stenotic on the sheet, a V-shaped incision of the sheet margin surrounding the ICA was made and the tension of the sheet was released.

## 3. Results

### 3.1. Patient demographics

Characteristics of BBAs are summarized in Table 1. Eight (32%) patients had a history of hypertension. The female/male ratio was 19/6, demonstrating a strong female predominance. The mean patient age was 39.5 ± 11.3 years. All 25 patients presented with SAHs caused by rupture of BBAs (Fisher grade 2 in 1 patient, grade 3 in 14 patients, and grade 4 in 10 patients). The mean duration of clinical follow-up was 128.9 months (range, 85–196 months). Three patients were lost to follow-up. One patient was lost to follow-up due to emigration. Two patients were lost after 8 years due to unknown causes.

**Table 1:** Summary of characteristics in 25 patients with BBAs.

	Age/sex	Preop H–H/Fisher	Premature rupture	Op method	Neck suture	Carotid stenosis	12 mo mRS
1	24/M	2/2	No	CR		No ICA stenosis	0
2	48/F	3/3	Yes	CR		Mild stenosis	1
3	46/F	3/4	Yes	CR	Yes	Moderate stenosis	3
4	51/F	3/4	Yes	CR		No ICA stenosis	2
5	47/F	4/3	No	CR		Mild stenosis	1
6	49/F	3/3	Yes	CR		Mild stenosis	1
7	51/F	3/3	No	CR		Mild stenosis	4
8	46/F	2/3	Yes	CR		Mild stenosis	3
9	59/M	2/4	No	CR		Mild stenosis	2
10	42/M	4/3	No	CR		Moderate stenosis	2
11	37/F	2/3	No	CR		Mild stenosis	0
12	58/F	3/4	No	CR		No ICA stenosis	1
13	60/F	3/4	No	CR		No ICA stenosis	1
14	39/F	3/3	Yes	CR	Yes	Moderate stenosis	1
15	29/M	3/4	Yes	CR	Yes	Total occlusion	3
16	45/F	4/4	No	DC		Mild stenosis	2
17	51/F	3/4	No	DC		No ICA stenosis	1
18	38/F	3/4	No	CR		Mild stenosis	1
19	33/M	3/3	Yes	DC	Yes	Mild stenosis	0
20	45/F	2/3	Yes	DC		No ICA stenosis	0
21	47/F	3/4	Yes	CR	Yes	Moderate stenosis	1
22	45/F	2/3	No	DC		No ICA stenosis	2
23	46/F	3/4	No	DC		No ICA stenosis	1
24	66/F	2/3	No	CR		Moderate stenosis	1
25	61/F	4/3	Yes	CR	Yes	Moderate stenosis	2

BBAs = blood blister like aneurysms, CR = clip reinforcement, DC = direct clip, Fisher = Fisher scale, H–H = Hunt and Hess scale, mRS = modified Rankin Scale, Op method = operation method, Preop = preoperative.

Five (20%) patients presented with multiple aneurysms. Six (24%) patients had chronic post-hemorrhagic hydrocephalus requiring ventriculoperitoneal shunts. The interval from ictus to surgical treatment was 2.7 ± 12.5 days. The SAH was classified using the Hunt and Hess grading system: 4 (32%) patients with grade 2, 13 (52%) with grade 3, 4 (16%) with grade 4. Nine (36%) patients had SAHs combined with intraventricular hemorrhage. One patient had intraventricular and intracerebral hematomas in the frontal lobe, and 1 patient had a subdural hematoma along the falx.

### 3.2. Aneurysm characteristics

All aneurysms were located in the supraclinoid portion of the ICA. The mean aneurysm diameter was 4.04 ± 1.3 mm. Twenty-one (84%) aneurysms were ≤5 mm in diameter. BBAs were localized on the right side in 11 patients and on the left side in 14 patients. Aneurysmal directions in the operative field are listed in Table 1. The direction of the aneurysm was dorsomedial in 15 patients and dorsal in 10 patients. All patients underwent conventional 4-vessel CAG or DSA. For 2 patients, the initial conventional CAG failed to confirm aneurysms, which were seen on the second CAG. After 14 days of follow-up, the CAG revealed an increased size of the aneurysm (3 mm).

### 3.3. Surgical results

The mean time from ictus to surgery was 2.7 days. Intraoperative microscopic findings were confirmed in all patients. Before treatment, 4 (16%) episodes of recurrent bleeding occurred in 4 patients within 3 days after the initial bleeding. Aneurysms were treated by direct clipping with a Bemsheet^®^ in 5 (20%) patients or clip reinforcement with a silicone sheet in 20 (80%) patients with intention of parent artery preservation. Eleven (44%) patients experienced intraoperative premature rupture during dissection. Tearing of the aneurysmal neck during dissection occurred in 8 (32%) patients (Fig. 2B). Six of 8 patients with neck tearing underwent direct neck repair with nylon 8-0 (Fig. 2C). Clip reinforcement was then performed (Fig. 2D). For proximal control during aneurysmal clipping, 7 patients were exposure to the cervical carotid artery. The overall mean time of temporary clipping time was 11.4 minutes (range, 0–43 minutes). The mean time in the directing clipping group was 3 minutes (range, 0–12 minutes). In the patients with premature rupture, it was 26.7 minutes (range, 12–43 minutes). In patients with neck tears treated by direct neck repair, it was 32.5 minutes (range, 15–43 minutes). All patients had confirmed preservation of the parent artery using intraoperative microvascular Doppler. Symptomatic vasospasm developed in 10 (40%) patients. After hypertensive hypervolemic hemodilution therapy failed in 1 patient, endovascular treatment with percutaneous balloon angioplasty and intra-arterial papaverine injection was performed. Radiological cerebral infarctions occurred in 5 patients (25% of the study population), including 2 patients with cerebral infarction presented with permanent hemiparesis and 3 patients with transient hemiparesis. All patients with cerebral infarction were treated with clip reinforcement. One of them had neurologic deterioration immediately after surgery because of a long period of temporary clip (42 minutes). For others, cerebral infarction occurred during the period of vasospasm, two of which occurred on the contralateral side of the surgical approach. All patients with cerebral infarction had mild-to-moderate parent artery stenosis. No patient had postoperative bleeding, clip sliding, or aneurysmal regrowth during the follow-up period.

### 3.4. Clinical follow-up outcomes

Clinical follow-up data of all patients were collected. The mean duration of clinical follow-up was 128.9 months (range, 85–196 months). Clinical outcomes were favorable (mRS of 0–2) in 21 (84%) of 25 patients. Four (16%) patients had an unfavorable outcome (mRS of 3–6). The patient with severe disability (mRS 4) was treated with clip reinforcement with direct neck repair. Six days postoperatively, the patient exhibited right hemiparesis with gradual mental deterioration and new lesions on subsequent CT scans. An immediate angiogram revealed severe ICA stenosis. Balloon angioplasty was performed without procedure-related complications. Two years later, the final angiogram revealed ICA occlusion with permanent neurological deficits. Overall management morbidity, including the aforementioned procedure-related morbidities, was 16%. No death occurred in this study.

### 3.5. Angiographic follow-up results

The final angiographic follow-up time was 87.6 months. Follow-up angiography revealed complete obliteration in all patients. The most important angiographic follow-up result was stenosis of parent artery. In the direct clipping group, there was no parent artery stenosis. However, in the clip reinforcement group, 17 (85%) of 20 patients had parent artery stenosis of various degrees. The degree of parent artery occlusion is often overestimated during parent-artery spasm. Thus, angiographic results were assessed at 14 days after surgery and at the last follow-up angiography.

**Figure 3.**
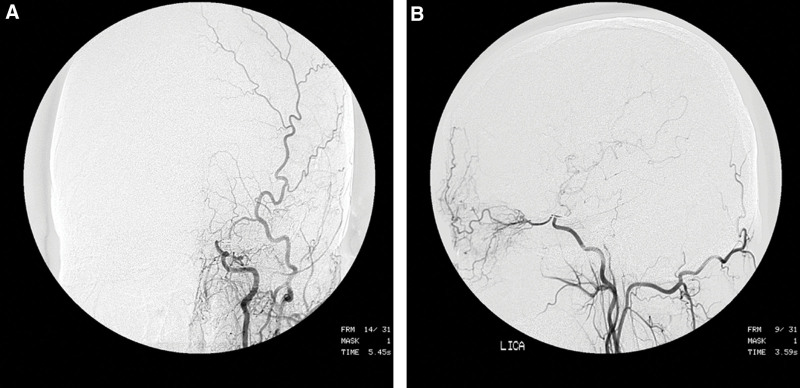
(A and B) Follow-up angiography obtained 9 months later demonstrating total occlusion of the left internal carotid artery just distal to the ophthalmic artery.

Postoperative supraclinoid stenosis of the ICA was categorized as normal, mild stenosis (<29%), moderate stenosis (30%–69%), severe stenosis (70%–99%), or total occlusion (99%–100%) as detailed by the North American Symptomatic Carotid Endarterectomy Trial criteria. In our series, mild stenosis in 10 (40%) patients, moderate stenosis in 6 (24%) patients, and total occlusion in 1 (4%) patient were confirmed (Fig. 3A and B).

## 4. Discussion

There are several approaches to the management of BBAs involving the supraclinoid portion of the ICA, including direct clipping, clip reinforcement, bypass with trapping, reconstructive endovascular coiling, stent-assisted coiling, covered stent, and endovascular trapping. The appropriate treatment of ICA BBAs remains controversial.

Herein, we report the safety of surgical treatment for ICA BBAs and the long-term efficacy of prevention of recurrent bleeding and re-growth after SAH. In agreement with previous reports,^[9,10,14,27,33–37]^ our results of surgical treatment for ICA BBAs were satisfactory with an acceptable, treatment-related morbidity rate.

### 4.1. Endovascular treatment

Endovascular treatment provides an alternative to surgery for the management of ruptured BBAs. However, BBAs embolized with coils may regrow and rebleed.^[16–18,21,22,25,38]^ Results of the present study showed that stent-assisted coil embolization was inadequate in preventing BBA regrowth. Thus, stent placement within a stent or covered stent placement should be considered as an alternative treatment for BBAs.

The use of flow-diverting stents to treat ruptured BBAs is associated with high rates of complete occlusion and good long-term neurological outcomes in most patients in a previous study.^[19,26]^ When 10 patients with ruptured BBAs of the supraclinoid ICA were treated using a pipeline embolization device, the placement of a single pipeline embolization device resulted in an immediate occlusion or near occlusion of the BBA in 9 of 10 patients. Follow-up DSA showed complete occlusion of the BBA.^[19]^

### 4.2. Surgical treatment

The most important characteristic of BBAs is the very high rate of premature rupture, BBA avulsion, and ICA lacerations during surgery due to a fragile aneurysmal wall.^[2,3,7,14,29]^ BBAs avulse easily when the clip is applied regardless of a careful placement of blades parallel to the axis of the ICA. Avulsion of the aneurysmal neck has a very poor prognosis.^[34,36]^ Ogawa et al have reported surgical results of ICA BBAs. Forty patients with ICA BBAs had markedly poorer clinical outcomes than those with saccular-type aneurysms.^[36]^ Postoperative rebleeding occurred in 8 patients treated with direct clipping or wrapping.^[36]^ All these patients died. They concluded that clipping or wrapping alone could not prevent rebleeding in patients with ICA BBAs and that clipping of wrapping material should be used. Recently, Meling and Patet^[14]^ reported results of 6 patients with BBAs who underwent surgical treatment, including the Gore-Tex clip-wrapping technique without intraoperative rupture or parent artery sacrifice with good results. In our study showed, when surgical and endovascular risks of BBAs were compared, clip reinforcement was a safe and effective surgical method despite a parent artery stenosis.

Considering difficulties involved in achieving complete clip occlusion of ICA BBAs and the relatively high rate of premature rupture, BBA avulsion, and ICA lacerations attributable to direct surgery, clip reinforcement with or without neck suture and extracranial-intracranial bypass surgery have become favored therapeutic methods during acute surgery.^[9]^

Technical developments in neurosurgery have been significant. The use of direct neck repair and clip reinforcement to repair a tear at the base of an aneurysm is essential. The repair of the arterial tear in the ICA can be very difficult because the operative field is deep and narrow in the acute stage. For this reason, this surgical technique requires more time for a temporary clip than for a direct clip. In this situation such as aneurysmal neck tearing, direct neck repair must be performed despite a long period is required for a temporary clip. We placed three or four 8-0 nylon microsuture stitches in the ICA to reform the parent artery rather than completely suture the tear. It is unnecessary to completely obliterate the arterial wall defect.^[37,39,40]^ Three or 4 stitches were sufficient to obliterate arterial wall defects. Therefore, the temporary occlusion time can be shortened considerably. To reduce the period of a temporary clip, surgeons should prepare to cope with all possible situations, such as bypass or direct neck suture. Before Sylvian fissure dissection, superficial temporal artery—middle cerebral artery bypass surgery is an alternative method to prevent ischemia during long periods of temporary clips.

BBAs are treated by wrapping with a silicone sheet in a circumferential manner. The primary advantage of silicone sheets is that they are thin, flexible, and transparent. One of the advantages of a silicone sheet is that the encircling silicone sheet is transparent, which makes it easy to observe structures underneath it after clip application. Branches around the aneurysm encircled with the sling can also be seen. In our study, the risk of rebleeding was less than other treatment options such as endovascular coiling or endovascular trapping of the ICA. The long-term efficacy of surgical treatment was assessed. Our study showed promising results. Our results suggest that surgical treatment is the preferred treatment for patients with ruptured BBAs.

When performing clip reinforcement with or without direct neck suture during surgery, the most worrying thing is ICA stenosis. Sometimes this is intentional when the ICA is sacrificed as the chosen therapy for aneurysm obliteration. However, this maneuver is clearly associated with the potential of ischemic complications when it is performed in an unplanned and uncontrolled manner. Delayed cerebral infarction is an important cause of death and disability after SAH. Recently, Meling and Strickland have shown the effect of extracranial-to-intracranial bypass surgery with parent vessel trapping for BBAs of the ICA.^[41,42]^ They concluded that extracranial-to-intracranial bypass with ICA trapping might be a valid treatment option for ruptured ICA BBAs.^[41,42]^

The rate of cerebral infarction caused by vasospasm ranges from 24% to 35% when defined by CT. It might be as high as 81% when magnetic resonance imaging is used for diagnosis.^[43,44]^

Theoretically, in immediate postoperative and vasospasm periods, stenosis of the parent artery is a risk factor for cerebral ischemia or infarction. However, in our study, ipsilateral cerebral infarction occurred in 2 patients with total occlusion and mild stenosis of the parent artery. Consequently, the incidence of delayed cerebral infarction in patients with carotid stenosis was not high compared to that in the non-stenotic group.^[45]^ Postoperative carotid stenosis may play a role as risk factor for cerebral infarction. However, mild-to-moderate stenosis of the parent artery did not increase the risk of cerebral ischemia or infarction during the vasospasm period. The fear of postoperative stenosis dose not exert sufficient tension on the parent artery. Postoperative catastrophic results such as rebleeding or regrowth might occur. In our experience, the most important aspect of clip reinforcement was sufficient tension despite carotid stenosis. Mild-to-moderate stenosis of the parent artery did not increase the risk of cerebral ischemia or infarction during vasospasm periods.

This study has some limitations. This is a single center experience and retrospective study. Treatments of BBAs are still controversial and difficult. Various surgical and endovascular treatments have been attempted in the past. Surgical treatment has been considered difficult due to the high fragility of the BBA wall, and it is associated with poor prognosis. Various endovascular treatment may be difficult due to the lesion’s wide neck and hemispheric dome with fragile BBA wall. A better understanding of BBAs and development of new therapeutic techniques is required.

## 5. Conclusion

Although surgical treatment of BBAs was associated with various degrees of patency loss in the parent vessel, long-term follow-up results of more than 10 years showed that direct surgical clipping or clip reinforcement with a silicone sheet appeared to be a more creative or curative surgery. However, the high rate of patency loss in the parent vessel and the long period of temporary clip are our challenges. Our experience reintroduces microsurgery as a safe and more durable treatment option for the management of ICA BBAs.

## Author contributions

**Writing – original draft:** Jong Myong Lee.

**Writing – review & editing:** Hyeon-Ju Kim, Jong Myong Lee.
